# 

**Published:** 1888-11

**Authors:** 


### The Chicago Medical Journal and Examiner.

Volume 57. Number 5.

### CONTENTS OF THIS NUMBER.

**Original Communications—** PAGE.

Twelve Months of Abdominal and Vaginal Section. H. T. Byford ........ 257

Pathology and treatment of a Dry Tongue. W. C. Caldwell ........ 268

**Editorial—**

Exercise and Fatty Heart ........ 277

The Need of a Medical Reference Library ........ 278

**Obituary—**

Professor Elkanah Williams, M. D. ........ 279

**Society Reports—**

Transactions of the American Association of Obstetricians and Gynæcologists ........ 279

Mississippi Valley Medical Association ........ 300

Chicago Pathological Society ........ 306

**Extracts and Abstracts** ........ 310

**Book Reviews and Notices** ........ 314

**Miscellany** ........ 319

**Announcements** ........ 320

**Index to Advertisers** ........ x

